# Case Report: A multidisciplinary collaborative case of complex rectovaginal fistula resulting from childhood sexual trauma

**DOI:** 10.3389/fmed.2026.1753114

**Published:** 2026-02-24

**Authors:** Xin Du, Xuemei Sun, Yujing Zhao, Xiuhua Fan

**Affiliations:** 1 Guang'anmen Hospital, China Academy of Traditional Chinese Medicine, Beijing, China; 2 Beijing Chaoyang District Maternal and Child Health Care Hospital, Beijing, China

**Keywords:** chronic grade IV perineal laceration, multidisciplinary treatment, psychological intervention, rectovaginal fistula, sexual trauma

## Abstract

Rectovaginal fistula (RVF) is a severe gynecological complication that can arise from obstetric trauma, surgical injury, infection, or physical trauma. RVF resulting from childhood sexual assault accompanied by extensive perineal injury is uncommon in clinical practice, and its management is particularly complex, as it requires not only meticulous anatomical reconstruction but also sustained attention to long-term psychological sequelae. This report presents the case of a patient with RVF secondary to childhood sexual assault. The patient underwent two unsuccessful repair attempts and subsequently developed a complex RVF associated with an old grade IV perineal laceration. At presentation, she reported intermittent passage of fecal material through the vagina. Physical examination showed a complete loss of the perineal body and central tendon, along with the absence of the rectovaginal septum. Pelvic magnetic resonance imaging revealed an incomplete posterior vaginal wall measuring 3.4 cm, extending from the lower vaginal segment to the vaginal introitus, consistent with RVF. Definitive anatomical reconstruction was achieved through close intraoperative collaboration among gynecology, colorectal surgery, and anesthesiology. Comprehensive perioperative psychological evaluation and psychiatric support alleviated trauma-related distress, improved treatment adherence, and facilitated postoperative recovery. Specialized nursing care optimized perioperative management and supported functional rehabilitation. Within this multidisciplinary team (MDT) framework, a successful single-stage surgical repair was accomplished. This case highlights the value of an individualized, MDT-based approach that integrates surgical reconstruction with psychological intervention in the management of complex RVF. Despite previous failed repairs and delayed intervention, a coordinated single-stage MDT strategy resulted in a favorable outcome. We further analyze the potential causes of previous surgical failure, identify key determinants of successful repair, and provide practical insights to guide the management of similar complex cases in clinical practice.

## 1 Introduction

Rectovaginal fistula (RVF) and perineal laceration are traumatic disorders involving the female pelvic floor and the perineum. RVF is defined as an abnormal communication between the rectum and the vagina, leading to the passage of feces or gas through the vaginal canal rather than the anus. It represents a pathological connection between the digestive and reproductive systems and typically presents with the vaginal passage of flatus or fecal material (1). Perineal laceration refers to the disruption of the perineal skin, mucosa, and underlying musculature, including the superficial transverse perineal muscles and the anal sphincter complex. It is most often caused by external traction or traumatic injury (2). Based on the severity and anatomical extent of tissue damage, perineal lacerations are classified into grades I–IV, with grade IV constituting the most severe form. Fourth-degree perineal lacerations involve complete rupture of the external anal sphincter and levator ani muscles, accompanied by injury to the rectal mucosa, resulting from concurrent tearing of the anterior rectal wall and the posterior vaginal wall. Due to significant sphincter disruption, fecal incontinence is a prominent clinical feature. RVF and severe perineal lacerations are closely related in terms of etiology, pathophysiology, clinical manifestations, and therapeutic outcomes, particularly in the context of obstetric trauma. However, RVF resulting from childhood sexual assault combined with a long-standing fourth-degree perineal laceration is rarely reported and presents substantial challenges in clinical management.

**Figure 1:**
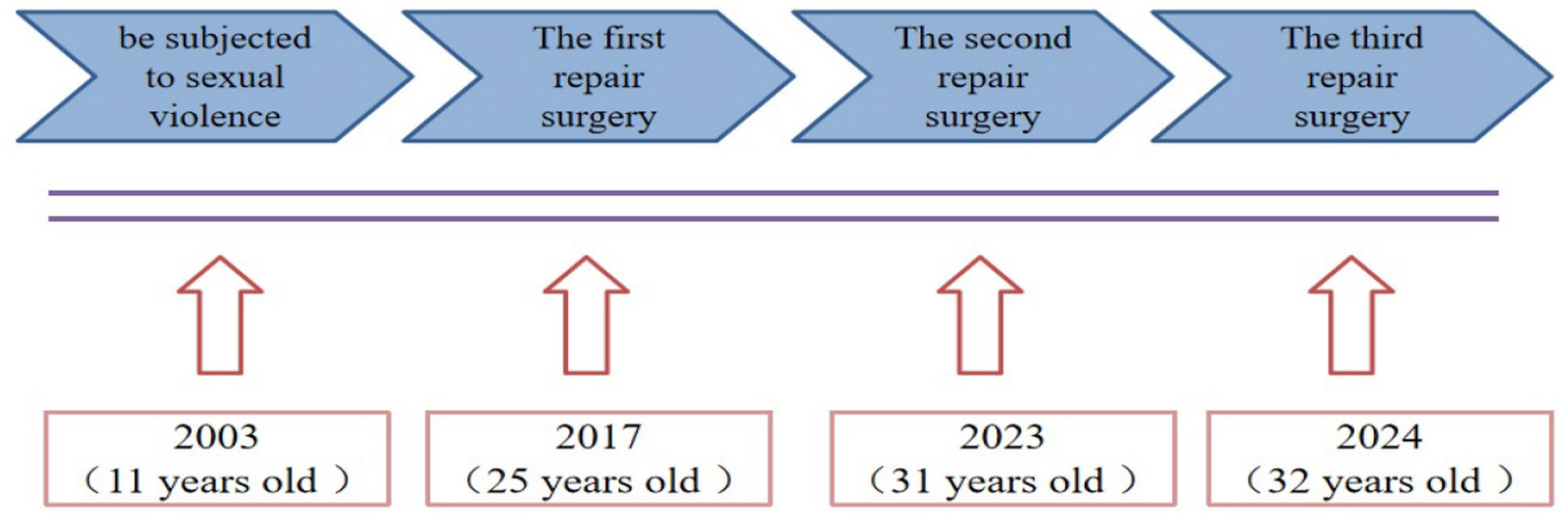
Timeline of historical and current episode of care the patient experienced sexual assault at 11 years of age (2003), resulting in rectovaginal fistula and severe perineal injury. The first and second repair surgeries were performed at 25 years (2017) and 31 years (2023), respectively, both with incomplete symptom resolution. Definitive multidisciplinary single-stage repair was performed at 32 years of age (2024).

This report presents a patient who developed RVF and an old fourth-degree perineal laceration following childhood sexual assault. Despite two surgical repair attempts over 14 years, including a second procedure performed 6 years after the initial intervention, complete anatomical restoration was not achieved (Figure 1). This case illustrates the considerable diagnostic and therapeutic complexity associated with delayed presentation and repeated surgical failure in the management of complex RVF. Moreover, it underscores the critical importance of addressing psychological trauma in parallel with anatomical and functional reconstruction. Systematic psychological evaluation and targeted intervention are integral components of comprehensive care for both patients and their families. A holistic, mind–body–integrated management strategy may improve the quality of life, support the overall rehabilitation, and provide a crucial foundation for the successful execution of single-stage surgical repair.

## 2 Case report

A 32-year-old woman reported a history of sexual assault at 11 years of age, after which she developed a rectovaginal fistula (RVF). She subsequently underwent two surgical repair procedures at 25 and 31 years of age; however, symptom resolution was incomplete, and fecal incontinence persisted. At the time of admission, the patient complained of abnormal vulvar appearance that adversely affected her marital quality of life, inability to control loose stools, and intermittent passage of fecal material through the vagina. Owing to her partner’s failure to accept the vulvar appearance, she underwent two medical abortions following unintended pregnancies. The patient also reported long-standing psychological distress accompanied by anxiety and depressive symptoms and sought vulvar reconstruction to improve her overall quality of life.

**Figure 2:**
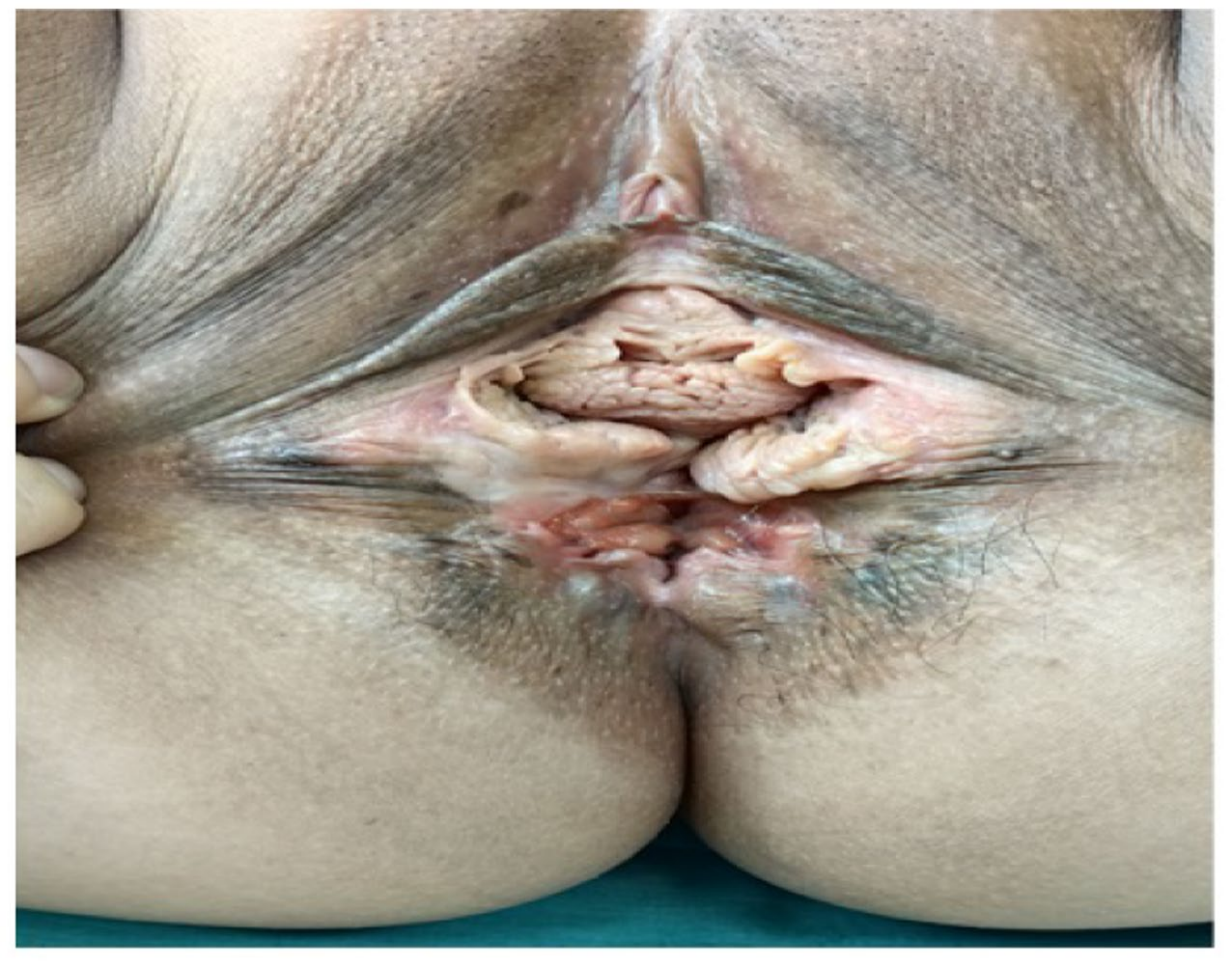
Preoperative perineal appearance. Preoperative examination revealed severe perineal defect, with near-complete absence of the perineal body and central tendon, exposure of rectovaginal communication, and marked distortion of normal perineal anatomy, consistent with an old fourth-degree perineal laceration.

**Figure 3:**
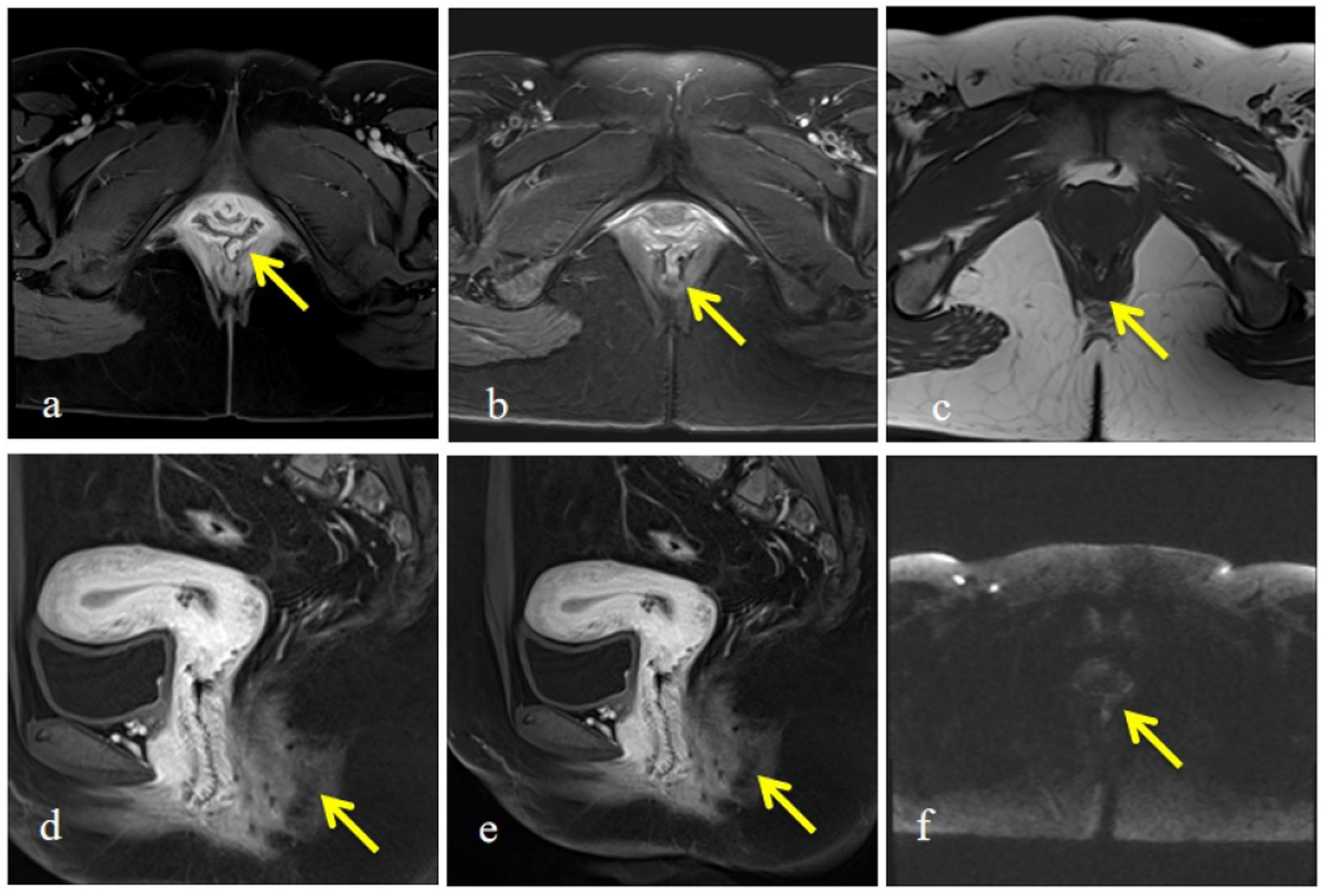
Pelvic magnetic resonance imaging demonstrating rectovaginal fistula. **(a)** Contrast-enhanced axial image showing disruption of the rectovaginal septum with abnormal communication between the rectum and vagina (arrow). **(b)** T2-weighted axial image demonstrating hyperintense fistulous tract connecting the rectum and vaginal lumen (arrow). **(c)** T1-weighted axial image revealing structural discontinuity of the posterior vaginal wall (arrow). **(d)** Contrast-enhanced sagittal image showing fistula tract extending from the rectum to the posterior vaginal wall (arrow). **(e)** Sagittal contrast-enhanced image further delineating the fistulous pathway and surrounding soft tissue changes (arrow). **(f)** Diffusion-weighted imaging highlighting the fistulous region with abnormal signal intensity (arrow).

On gynecological examination, the vulva was underdeveloped, with a complete absence of the perineal body and the central tendon. The rectovaginal septum was not identifiable, and a mild protrusion of the anterior vaginal wall was observed. A 3-cm midline tear was present at the 6 o’clock position of the posterior vaginal wall, extending into the rectal mucosa. The vagina communicated directly with the anus, with exposure of the rectal lumen and visible fecal residue at the vaginal introitus. A bimanual examination revealed an anteverted uterus of normal size, with no palpable abnormalities in either adnexa. An anorectal examination showed significantly reduced anal sphincter tone, and a digital rectal examination identified a palpable fistulous tract involving the rectum and the anal canal (Figure 2). Pelvic magnetic resonance imaging (MRI) further confirmed the presence of an RVF (Figure 3). Based on these findings, a diagnosis of RVF associated with an old grade IV perineal laceration was established. Following a multidisciplinary discussion, surgical repair of the RVF, combined with perineoplasty, was planned.

After admission, comprehensive laboratory testing and imaging evaluations were completed. In addition to standard preoperative preparation, adjunctive traditional Chinese medicine interventions were initiated to optimize the patient’s perioperative psychological status. Acupuncture was administered at Baihui (GV20), Shenmen (HT7), Neiguan (PC6), Hegu (LI4), Taichong (LR3), and Sanyinjiao (SP6). This was combined with five-element music therapy, emphasizing the Jue tone, corresponding to the liver in traditional Chinese medicine theory, to alleviate psychological stress, regulate qi and blood circulation, and improve emotional stability. These integrative measures were used as supportive interventions to improve surgical tolerance and postoperative recovery.

Colorectal surgery, psychiatry, and anesthesiology revealed no contraindications to surgery, and the patient was deemed suitable for operative intervention. The surgical risks and potential complications were explained in detail to the patient and her family, who provided written informed consent. Oral intestinal antibiotics (metronidazole tablets, 0.5 g per tablet, twice daily) were administered for 3 days before surgery.

The patient was placed in the lithotomy position, and the surgical field was routinely disinfected and draped. A urinary catheter was inserted, and sterile dry cotton balls were placed proximal to the rectum to prevent fecal contamination of the operative field.

**Figure 4:**
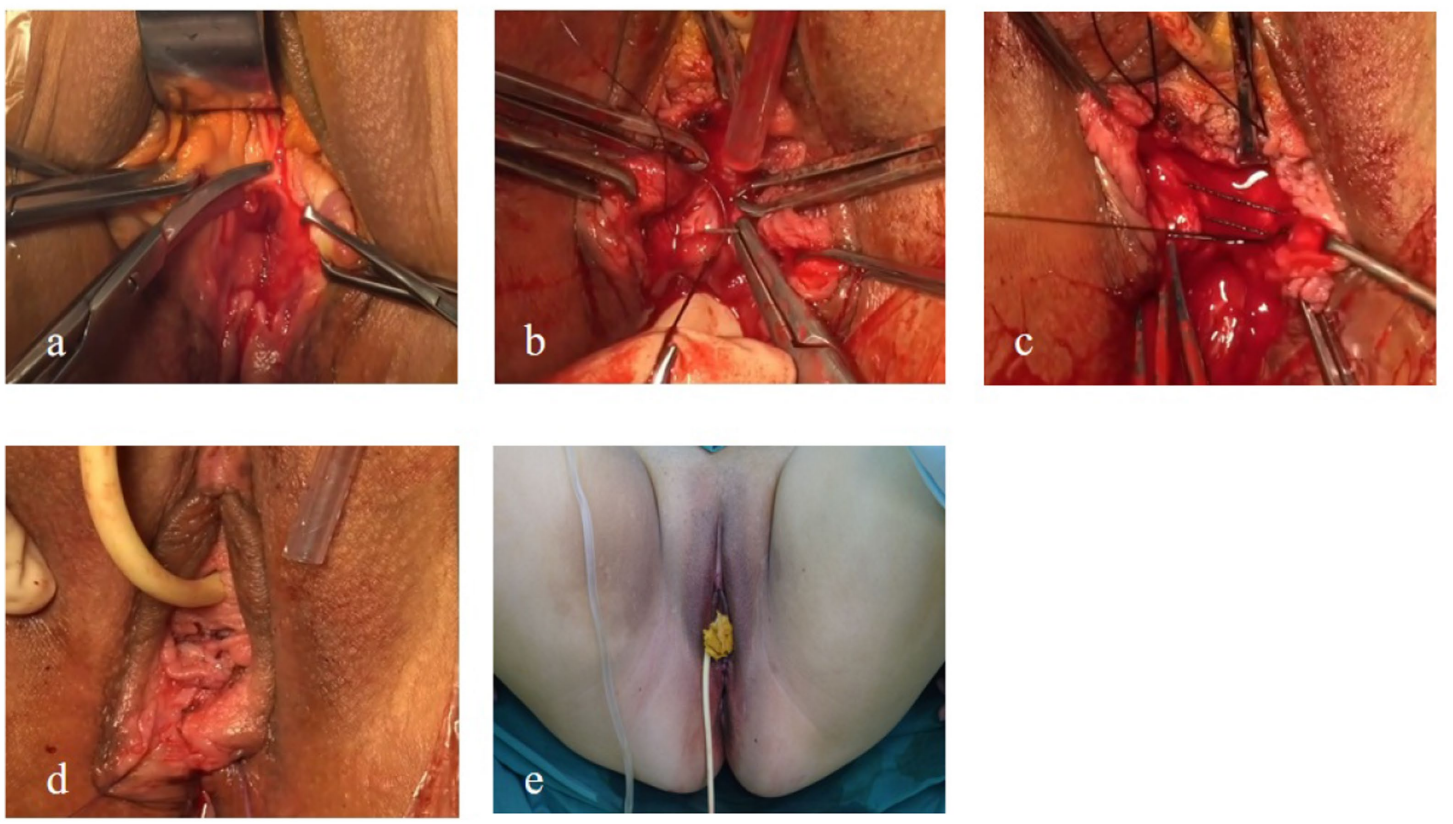
Intraoperative findings and key surgical steps. **(a)** Exposure of the operative field after excision of dense scar tissue within the rectovaginal space. **(b)** Full-thickness interrupted suturing of the rectal wall without penetration of the rectal mucosa. **(c)** Reconstruction of the levator ani muscle to restore pelvic floor support. **(d)** Formation of a new perineal body following layered anatomical repair. **(e)** Immediate postoperative appearance demonstrating restored perineal anatomy.

(a) Debridement and exposure: Two Allis forceps were applied to the lower margins of the old laceration on both sides of the hymenal ring, and two additional Allis forceps were used to grasp the torn edges of the rectovaginal wall. Scar tissue along the wound margins was excised to expose the junction between the vaginal wall and the rectum. Meningeal scissors, with the curved blade oriented upward, were used to separate the vaginal wall from the rectum within the rectovaginal space. The lateral margins of the vaginal flap were dissected to the most distal points on both sides of the hymenal scar.

(b) Repair of the rectal mucosa and the muscularis: The vaginal wall was incised to the appropriate depth in the midline. The vaginal mucosal flap was dissected laterally to expose the rectal wall, the levator ani muscles, and the sphincter ends. Dense scar tissue was excised at the rectal defect. Interrupted sutures using 3–0 absorbable material were placed at 0.5-cm intervals to repair the rectal wall, taking care not to penetrate the rectal mucosa.

(c) Anal sphincter repair: The sphincter ends were identified within the bilateral depressions, grasped using the Allis forceps, approximated toward the midline, and sutured using two 7–0 silk sutures, resulting in the contraction of the perianal skin folds into a ring-like configuration. The rectal fascia and the levator ani muscles were subsequently sutured using 2–0 absorbable sutures.

(d) Perineal body and vaginal reconstruction: Excess vaginal mucosa was excised. The lowest margin of the hymenal ring was grasped using the Allis forceps and drawn toward the midline. The reconstructed vaginal caliber was adjusted to permit the passage of two fingers, determining the height of the newly formed perineal body. The vaginal mucosa was closed using 2–0 absorbable sutures, followed by layered closure of the perineal subcutaneous tissue. The perineal skin was sutured using 3–0 absorbable material. Restoration of the anterior vaginal wall without protrusion was confirmed. All intraluminal cotton balls were removed entirely, and half a piece of iodine-impregnated gauze was placed in the vagina and removed 24 h postoperatively (Figure 4).

Postoperatively, prophylactic antibiotic therapy with intravenous levofloxacin sodium combined with piperacillin–tazobactam sodium was administered. Standard wound care and meticulous perianal hygiene were maintained to minimize the risk of infection. The patient was instructed to remain on strict bed rest and to abstain from oral intake, and the urinary catheter was retained. One week after the surgery, ambulation was permitted. Stool consistency remained soft, and wound healing was satisfactory. The urinary catheter was removed, and oral intake was gradually advanced from liquids to semi-liquids and then to solid foods. Throughout the perioperative period, acupuncture and adjunctive traditional Chinese medicine interventions were continued for emotional regulation and psychological support.

**Figure 5:**
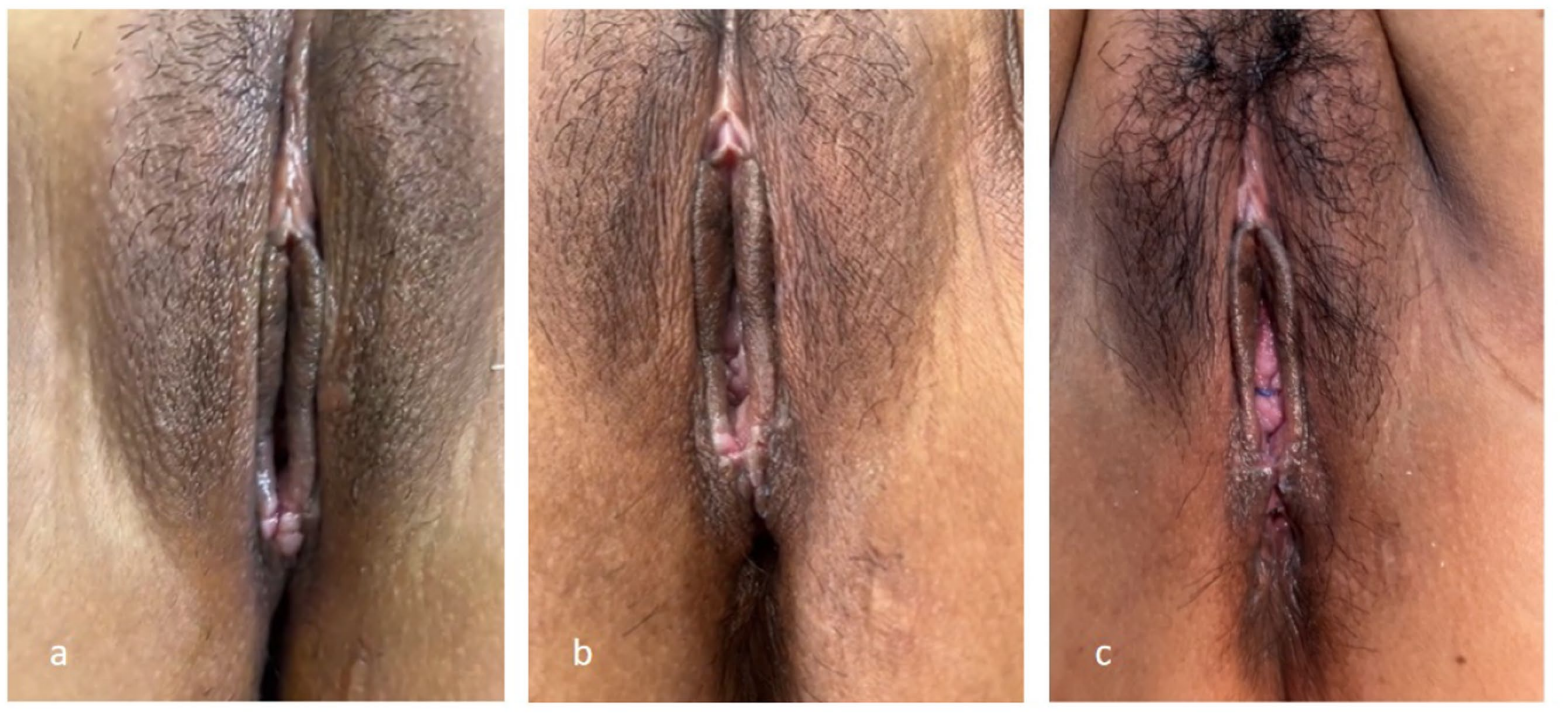
Postoperative perineal appearance during follow-up: **(a)** One week postoperatively, showing intact wound edges and satisfactory early healing; **(b)** two weeks postoperatively, demonstrating progressive wound healing and restoration of perineal contour; **(c)** One month postoperatively, showing well-healed perineum with stable anatomical reconstruction.

At 2 weeks postoperatively, a gross examination revealed regular perineal and anal contours with continuous skin, and the sutures had spontaneously dissolved. A digital rectal examination revealed well-healed wounds and strong anal sphincter contraction, with no additional abnormalities. At 1 month postoperatively, the patient reported normal defecation with good control of both solid and liquid stools (Figure 5). At the 6-month follow-up, she reported no discomfort but perceived the reconstructed perineal body to be relatively short. After careful discussion, considering the delayed intervention, previous reconstructive procedures, and intraoperative findings, the final reconstructed perineal body length was approximately 1 cm. The patient was advised to abstain from sexual activity for 6 months after discharge and to undergo cesarean delivery for any future pregnancies. We have maintained ongoing online communication with the patient, who, to date, reports normal bowel function and an unimpaired daily life without related complaints. However, she has not yet entered a romantic relationship, which currently precludes evaluation of her sexual function. Regular long-term follow-up will be continued in the subsequent period.

## 3 Discussion

The RVF and severe perineal lacerations most frequently arise from obstetric trauma, particularly prolonged labor, instrumental delivery, and fourth-degree perineal tears (2, 3). In comparison, RVF resulting from childhood sexual assault is exceedingly rare and remains sparsely documented in the literature (4). Such cases are often marked by delayed diagnosis and intervention, prolonged physical morbidity, and profound psychological consequences. In this case, the sexual assault occurred during childhood, and definitive surgical management was deferred for more than a decade. Previous studies have shown that delayed repair is associated with extensive fibrosis, compromised tissue vascularity, and increased technical difficulty, all of which can substantially elevate the risk of surgical failure (5). These factors likely contributed to the patient’s unsuccessful outcomes in the first two repair attempts.

The timing of surgical intervention is a critical determinant of RVF repair success. Reported success rates for primary RVF repair range from 70 to 90%; however, the outcomes are significantly poorer in complex or traumatic fistulas, particularly when dense scarring, ischemia, or previous failed repairs are present (6, 7). Current consensus generally recommends postponing surgical repair until local inflammation has resolved and tissue quality has improved, typically after a 3–6 month interval (3, 5). However, in cases involving childhood trauma, some authors advocate for earlier intervention, citing superior healing capacity and the potential to mitigate long-term psychological burden (4). In this patient, prolonged delay and repeated failed surgeries resulted in severe loss of the perineal body and sphincter dysfunction, significantly increasing the complexity of reconstruction.

The management of complex RVF necessitates a multidisciplinary team (MDT) approach. Accumulating evidence has revealed that optimal outcomes are achieved through close collaboration among gynecologists, colorectal surgeons, anesthesiologists, and mental health professionals (8). In this case, gynecology focused on vaginal reconstruction and perineoplasty, colorectal surgery addressed rectal wall and anal sphincter repair, and psychiatry provided comprehensive psychological evaluation and perioperative support. Such coordinated care not only improves anatomical and functional outcomes but also improves patient satisfaction and long-term quality of life (3, 8).

Psychological intervention is an essential and inseparable component of comprehensive management for RVF resulting from childhood sexual trauma. Psychological care should extend beyond the patient to include family members, who play a pivotal role in shaping disease perception, providing emotional support, and participating in treatment-related decision-making. Structured psychological counseling and effective communication with family caregivers can alleviate trauma-related stress, reduce misunderstanding, and establish a stable support network, thereby improving treatment adherence and facilitating perioperative and long-term recovery.

Previous studies have shown that childhood sexual abuse is associated with persistent psychological sequelae, including anxiety, depression, post-traumatic stress disorder, and disturbances in interpersonal relationships (8–10). These psychological impairments often interact with prolonged disease-related suffering, repeated treatment failure, and inadequate social support, resulting in significant deterioration in the quality of life and social functioning. Trauma-focused cognitive behavioral therapy and eye movement desensitization and reprocessing are among the most widely used evidence-based psychological interventions and have been shown to alleviate trauma-related symptoms and improve emotional regulation (11). However, in patients experiencing chronic and complex psychological distress, multimodal and individualized integrative strategies are frequently required.

Compared with conventional psychological interventions alone, traditional Chinese medicine (TCM) offers potential advantages through its holistic perspective, multi-target regulation, and favorable tolerability in the management of emotional disorders. Acupuncture, a core modality of TCM, is grounded in meridian theory and modulates qi and blood circulation through the stimulation of specific acupoints. Contemporary studies have suggested that acupuncture may exert antidepressant and anxiolytic effects by influencing monoaminergic neurotransmission, attenuating inflammatory responses, regulating hypothalamic–pituitary–adrenal axis activity, enhancing synaptic plasticity, and upregulating brain-derived neurotrophic factor expression (12, 13).

Five-element music therapy (FEMT) (14), derived from traditional five-element theory, integrates musical stimulation with the correspondence among the five tones, five zang-organs, and five emotions. In TCM theory, emotional dysregulation is closely associated with internal organ function, with liver qi stagnation commonly associated with depressive states. FEMT uses specific musical tones to restore emotional balance; the Jue tone, corresponding to the liver, is traditionally used to relieve emotional tension and promote psychological stability. Existing studies have indicated that music-based interventions can modulate cortical activity, induce relaxation, reduce stress responses, and improve emotional wellbeing, providing supportive benefits in the management of trauma-related psychological disorders.

In this case, psychological management was implemented throughout the perioperative period, integrating systematic psychological assessment with adjunctive acupuncture and FEMT while also providing appropriate psychological guidance to family members (15). This patient- and family-centered, multimodal supportive strategy was associated with improved emotional stability, improved treatment cooperation, and a favorable perioperative experience. By addressing psychological trauma at both individual and familial levels, this integrative approach created optimal conditions for successful single-stage surgical repair, postoperative functional recovery, and reintegration into daily life and social functioning.

From a surgical standpoint, several technical principles are essential for the successful repair of complex RVF. Comprehensive preoperative assessment, including pelvic MRI, is necessary to accurately define fistula location, size, and surrounding tissue status (3, 5). Complete excision of fibrotic scar tissue is critical to establish a well-vascularized wound bed and reduce the risk of recurrence (6, 16). A layered closure of the rectal wall, the rectovaginal septum, and the vaginal wall helps minimize tension and promotes optimal healing (16). In patients with concomitant anal sphincter injury, simultaneous sphincter reconstruction has been shown to significantly improve postoperative continence and functional outcomes (7, 16).

Multiple surgical techniques have been described for RVF repair, including local tissue closure; advancement flap procedures, such as Martius or gracilis muscle flaps; staged surgery; and fecal diversion (3, 7, 17). Each technique has specific indications, advantages, and limitations, and no single approach is universally applicable. Thus, surgical strategy should be individualized based on fistula characteristics, tissue condition, previous surgical history, and patient-specific needs.

Local tissue repair is often considered a first-line option for low and simple RVF due to its relatively limited invasiveness and shorter operative time (18). However, the outcomes are less favorable in complex cases characterized by extensive scarring, compromised vascularity, or associated sphincter injury. Recurrence rates increase substantially following previous failed repairs, limiting the effectiveness of repeated local closure.

Advancement flap techniques, including Martius and gracilis muscle flaps, aim to improve healing by interposing well-vascularized tissue and are frequently recommended for recurrent or high-risk fistulas (19). However, flap-based reconstruction is associated with increased surgical trauma, longer operative duration, and potential donor-site morbidity, including additional scarring, sensory disturbances, or functional impairment. In patients in whom adequate mobilization of local tissue can be achieved after complete scar excision and who can meet functional demands without tissue transfer, routine flap interposition may impose an unnecessary surgical burden.

Staged surgical strategies typically involve temporary fecal diversion with sigmoid colostomy, followed by delayed fistula repair and subsequent stoma closure (7). Although fecal diversion can reduce contamination and improve local healing conditions in the presence of active infection, severe inflammation, or multiple previous failures, it is associated with considerable psychological distress, reduced quality of life, increased financial cost, and prolonged treatment duration. Evidence indicates that patients with a temporary stoma frequently experience anxiety, depression, and social adaptation difficulties, while stoma reversal introduces additional operative risks and potential complications.

In this case, despite a prolonged disease course and two prior failed repairs, comprehensive preoperative assessment confirmed well-controlled local conditions, absence of active inflammation or inflammatory bowel disease, and the technical feasibility of complete scar excision with sufficient tissue mobilization. These findings indicated that definitive anatomical repair could be achieved without adjunctive diversion or flap interposition. Within a multidisciplinary decision-making framework, alternative strategies, including advancement flap procedures and staged repair with fecal diversion, were carefully considered. Although such approaches may be beneficial in cases of ongoing inflammation, compromised tissue quality, or recurrent infection, they were deemed less appropriate for this patient, given the stable local tissue environment and the substantial physical and psychological burden associated with multistage interventions. Therefore, a single-stage surgical repair was chosen to achieve definitive anatomical reconstruction while minimizing additional surgical trauma, psychological stress, and time-related costs. The favorable functional recovery and sustained symptom resolution observed during follow-up further support the suitability of this individualized treatment strategy.

Long-term follow-up is essential for patients with complex RVF, particularly those with a history of childhood trauma. Although the reconstructed perineal body length in this patient was approximately 1 cm, functional outcomes were satisfactory. Given the compromised perineal anatomy and previous surgical history, cesarean delivery is strongly recommended for future pregnancies to prevent recurrence or further injury (3, 16). Follow-up should extend beyond anatomical evaluation to include assessment of bowel function, sexual health, psychological wellbeing, and reproductive counseling. Efficacy in the present case was evaluated using three measures: the Wexner score, psychological status assessed by the Hospital Anxiety and Depression Scale (HADS), and quality of life assessed using the World Health Organization Quality of Life–BREF (WHOQOL-BREF) (Table 1).

**Table 1:** Quantitative assessment using the Wexner score, Hospital Anxiety and Depression Scale (HADS-A and HADS-D), and WHOQOL-BREF demonstrated progressive improvement from the preoperative period to the postoperative assessment and the 6-month follow-up, reflecting enhanced bowel continence, reduced anxiety and depressive symptoms, and improved overall quality of life.

Measure		Preoperative	Postoperative	6-month follow-up
Wexner score		12	8	6
HADS-A		14	10	8
HADS-D		13	7	5
WHOQOL-BREF	Physical health domain	35.71	50	71.43
	Psychological health domain	31.25	56.25	59.38
	Social relationships domain	40	60	70
	Environment domain	25	54.17	66.67

## 4 Conclusion

Complex rectovaginal fistulas resulting from childhood sexual trauma are uncommon and present significant therapeutic challenges, particularly in patients with extensive perineal damage and a history of failed surgical interventions. In this case, the patient experienced sexual assault at 11 years of age, and definitive management was delayed for several years due to social and environmental constraints, leading to two unsuccessful repair attempts and prolonged physical and psychological distress. Despite these adverse circumstances, favorable functional recovery was ultimately achieved through a single-stage surgical repair performed within a multidisciplinary care framework.

The outcome of this case highlights the critical value of a multidisciplinary strategy that integrates gynecological and colorectal surgical expertise with comprehensive psychological support, giving equal importance to anatomical restoration and mental healthcare. Careful selection of surgical timing, meticulous layered reconstruction, and individualized long-term follow-up are essential for optimizing functional outcomes. The insights derived from this case may provide practical guidance for the clinical management of similarly complex rectovaginal fistulas. However, functional outcome assessment in the present case relied primarily on clinical examinations, patient-reported symptoms, and follow-up interviews, without the use of objective investigative modalities such as endoanal ultrasound and anorectal manometry, which represents a key limitation of this study.

## Data Availability

The original contributions presented in the study are included in the article/supplementary material; further inquiries can be directed to the corresponding author.
